# Non-linear associations between inflammatory biomarkers and *Mycoplasma pneumoniae*–associated necrotizing pneumonia in children: a restricted cubic spline analysis and nomogram development

**DOI:** 10.3389/fped.2026.1915170

**Published:** 2026-09-15

**Authors:** Jiafeng Zheng, Mengyao Zhang, Hanquan Dong, Bo Wang, Yongsheng Xu

**Affiliations:** Department of Pediatric Respiratory Medicine, Children’s Hospital, Tianjin University/Tianjin Children’s Hospital, Tianjin, China

**Keywords:** children, *Mycoplasma pneumoniae*, necrotizing pneumonia, nomogram, restricted cubic spline

## Abstract

**Background:**

To characterize non-linear associations between inflammatory biomarkers and *Mycoplasma pneumoniae*–associated necrotizing pneumonia (MPNP) in children using restricted cubic spline (RCS) analysis, and to develop a predictive nomogram.

**Methods:**

We retrospectively analyzed 319 children hospitalized for *Mycoplasma pneumoniae* pneumonia (MPP) at Tianjin Children's Hospital (2020–2025), comprising an MPNP group (*n* = 146) and *Mycoplasma pneumoniae* non-necrotizing pneumonia (MPNNP) group (*n* = 173). MPNP was defined on contrast-enhanced chest CT according to established pediatric criteria. Thirty-three pre-treatment variables were extracted. Candidate predictors were screened by least absolute shrinkage and selection operator (LASSO) logistic regression with 10-fold cross-validation. RCS analysis with non-linearity testing was applied to evaluate dose–response relationships, and a multivariable logistic regression nomogram was constructed. Model performance was assessed by ROC analysis, calibration plots, and decision curve analysis (DCA), with internal validation using 1,000 bootstrap resamples.

**Results:**

LASSO regression identified three independent predictors: white blood cell (WBC) count, C-reactive protein (CRP), and fever duration. RCS analysis revealed a significant non-linear, threshold-like association for WBC (*P* < 0.001; *P*-non-linearity = 0.014) and a significant non-linear, inverted U-shaped association for CRP (*P* < 0.001; *P*-non-linearity < 0.001), with risk peaking at intermediate concentrations and attenuating at very high levels, whereas fever duration showed an approximately linear positive relationship. The nomogram demonstrated strong discrimination in internal validation, with an apparent AUC of 0.941 (95% CI, 0.908–0.975) and a bootstrap bias-corrected AUC of 0.920 (95% CI, 0.877–0.962). Calibration curves closely approximated the 45° reference line, and DCA confirmed net clinical benefit across a broad range of threshold probabilities.

**Conclusions:**

WBC count, CRP, and fever duration independently predict MPNP, with WBC and CRP exhibiting distinct non-linear dose–response patterns. The RCS-based nomogram integrating these readily available predictors shows promising predictive performance, though external validation is required before clinical implementation.

## 1 Introduction

*Mycoplasma pneumoniae* (*M. pneumoniae*) is one of the most prevalent atypical bacterial pathogens responsible for community-acquired pneumonia (CAP) in school-aged children and adolescents worldwide, accounting for 10%–40% of pediatric CAP cases (1, 2). Although the clinical course of *Mycoplasma pneumoniae* pneumonia (MPP) is generally regarded as mild and self-limiting, an increasing proportion of affected children progress to refractory or severe disease, leading to a range of serious intrapulmonary complications including atelectasis, bronchiolitis obliterans, bronchiectasis, and necrotizing pneumonia (NP) (1, 3). Among these, *Mycoplasma pneumoniae*–associated necrotizing pneumonia (MPNP) has been recognized with increasing frequency in recent years, particularly in East Asian pediatric cohorts, and has emerged as an important cause of prolonged hospitalization, invasive procedures, and potential long-term pulmonary sequelae (4, 5).

The pathogenesis of MPNP is multifactorial, involving both direct cytotoxic injury by the organism and its metabolites, and—more critically—an excessive host immune-inflammatory response that leads to secondary lung damage (6). Upon *M. pneumoniae* infection, robust activation of the innate and adaptive immune systems triggers the release of large quantities of inflammatory mediators and cytokines, promoting local vasculitis, activation of the coagulation cascade, and microthrombus formation in pulmonary capillaries; these processes collectively result in ischemic necrosis and liquefaction of the lung parenchyma (7, 8). Clinically, this immunopathological mechanism is reflected by markedly elevated inflammatory biomarkers—such as white blood cell (WBC) count, C-reactive protein (CRP), and prolonged fever duration—in children who develop MPNP compared with those with non-necrotizing MPP (9, 10).

Early identification of children at risk for MPNP is of paramount clinical importance, as the diagnosis of NP on chest computed tomography (CT) is typically delayed, occurring a median of 16–25 days after symptom onset (5, 7). This diagnostic lag may result in missed opportunities for timely intervention. In response, several studies have sought to delineate risk factors for MPNP using conventional multivariable logistic regression and have identified elevated WBC, CRP, D-dimer, lactate dehydrogenase (LDH), and prolonged fever as significant predictors (9, 10). More recently, nomogram-based prediction models incorporating these clinical and laboratory variables have been developed to facilitate individualized risk estimation at the bedside (9, 10). However, the existing models share a common methodological limitation: they uniformly assume a linear relationship between continuous predictors and disease risk. In biological systems, inflammatory biomarkers may exhibit threshold effects, saturation phenomena, or other non-linear dose–response patterns that, if unaccounted for, can lead to model mis-specification and suboptimal risk estimation.

Restricted cubic spline (RCS) analysis offers a flexible statistical framework for modeling such non-linear associations while preserving interpretability within a multivariable regression context (11). Despite its growing adoption in clinical epidemiology, RCS has not yet been applied to characterize the dose–response relationships between inflammatory markers and MPNP risk in children.

To address this knowledge gap, the present study was designed with two objectives. First, we aimed to characterize the non-linear associations between key inflammatory biomarkers—specifically WBC count, CRP concentration, and fever duration—and the risk of MPNP in hospitalized children, using RCS analysis. Second, we sought to develop and internally validate a parsimonious RCS-based nomogram that integrates these readily available clinical predictors for individualized risk estimation. We hypothesized that WBC and CRP would exhibit distinct non-linear dose–response patterns with MPNP risk, and that incorporating these non-linear terms into a predictive model would yield superior discrimination and calibration compared with traditional linear modeling approaches.

## 2 Methods

### 2.1 Study design and participants

This retrospective study enrolled consecutive children hospitalized for MPP in the Department of Respiratory Medicine, Tianjin Children's Hospital, between 2020 and 2025. No matching by admission time, age, or sex was applied; all eligible patients were included sequentially. Because this study was restricted to inpatients admitted to three respiratory wards of a tertiary children's hospital, the enrolled MPNNP cases predominantly represented children with more severe non-necrotizing MPP requiring hospitalization, whereas milder cases managed on an outpatient basis were not captured. This admission-based sampling accounts for the relatively balanced ratio between the MPNP and MPNNP groups. MPP was diagnosed according to the Guidelines for the Diagnosis and Treatment of *Mycoplasma pneumoniae* Pneumonia in Children (2023 Edition) (12), requiring all of the following: (1) respiratory manifestations (fever, cough, wheezing, dyspnea, or rales); (2) compatible radiological findings; and (3) at least one microbiological criterion (see below).

MPNP was defined on contrast-enhanced chest CT according to established pediatric criteria (7), requiring all of the following features within an area of pulmonary consolidation: (1) multiple non-enhancing low-density regions reflecting parenchymal liquefaction and necrosis; (2) thin-walled cavities or small lucencies without a discernible wall; and (3) absence of rim enhancement at the cavity margins. Lung abscess—characterized by a solitary, thick-walled cavity with prominent peripheral rim enhancement—was specifically excluded.

Children were eligible if they met the diagnostic criteria for either MPNP or *Mycoplasma pneumoniae* non-necrotizing pneumonia (MPNNP) and fulfilled at least one microbiological indicator: a serum *M. pneumoniae* IgM titer ≥1:160, or a fourfold or greater rise in paired acute- and convalescent-phase sera; a positive *M. pneumoniae* polymerase chain reaction (PCR) in respiratory specimens (nasopharyngeal swab or sputum); detection of *M. pneumoniae* DNA by PCR in bronchoalveolar lavage fluid or pleural effusion; or high-copy-number *M. pneumoniae* sequences identified by targeted next-generation sequencing (tNGS). Assignment to the MPNP group additionally required confirmatory CT findings, whereas MPNNP controls showed no radiological evidence of necrosis throughout hospitalization and follow-up.

Children were excluded for congenital heart disease, immunodeficiency, connective tissue disease, readmission during convalescence from a pulmonary infection, congenital pulmonary infection, lung abscess, pulmonary cyst, congenital pulmonary sequestration, or other severe intrapulmonary complications (e.g., plastic bronchitis, bronchiolitis obliterans). A total of 319 children were included and stratified into the MPNP (*n* = 146) and MPNNP (*n* = 173) groups.

The study was approved by the Ethics Committee of Tianjin Children's Hospital (Approval No. 2024-LXKY-016). All records were de-identified before analysis, and the requirement for informed consent was waived given the retrospective design.

### 2.2 Data collection

Thirty-three demographic, clinical, and laboratory variables were retrospectively extracted from electronic medical records, including age, sex, weight, height, peak temperature, fever and cough duration, history of eczema or rhinitis, abnormal auscultation, unilateral involvement, atelectasis, pleural effusion, diminished breath sounds, and a panel of laboratory indices (WBC, neutrophil percentage, CRP, hemoglobin, platelets, procalcitonin, lactic acid, prealbumin, LDH, AST, ALT, CK, CK-MB, IL-6, IgE, ferritin, ESR, D-dimer, and fibrinogen). All laboratory values were obtained from the first blood sample collected after admission and before treatment. Handling of missing data. The overall proportion of missing data was low (< 5% for all variables). Missing values were imputed using multiple imputation by chained equations (MICE) with five imputation cycles under the assumption of missing at random (13, 14). A sensitivity analysis using complete case analysis confirmed that the results were materially unchanged. Handling of outliers. Continuous variables were screened for extreme values using the interquartile range (IQR) method, with values exceeding Q1−3 × IQR or Q3 + 3 × IQR flagged for review. All flagged values were cross-verified against original medical records; values confirmed as genuine clinical measurements were retained in the analysis to avoid selection bias. No data entry errors were identified.

### 2.3 Feature selection and model development

Continuous variables were compared using the Student's t-test or Mann–Whitney U test based on normality (Shapiro–Wilk test); categorical variables were compared using the chi-square or Fisher's exact test. To minimize overfitting and address multicollinearity, candidate variables were screened by least absolute shrinkage and selection operator (LASSO) logistic regression, with the optimal penalty coefficient (*λ*) selected via 10-fold cross-validation under the minimum binomial deviance criterion.

All continuous variables retained by LASSO were subsequently evaluated for potential non-linear associations with MPNP risk using RCS regression. As an initial step, each variable was fitted with four knots placed at the 5th, 35th, 65th, and 95th percentiles of its distribution, following the default quantile-based placement recommended by Harrell (15) and implemented in the rms package. For each variable, two likelihood ratio tests were performed: (1) an overall test evaluating whether the variable was significantly associated with MPNP risk (linear plus non-linear components combined), and (2) a non-linearity test comparing the full spline model against a reduced model containing only the linear term, to determine whether the non-linear components contributed significantly. If the non-linearity test was significant (*P* < 0.05), the variable was retained in its spline form; if non-significant, the variable was simplified to a linear term in the final model. For variables retaining spline terms, the optimal number of knots was further evaluated by comparing models with three, four, and five knots using the Akaike Information Criterion (AIC), selecting the most parsimonious specification that adequately captured the observed dose–response relationship. Reference values odds ratio (OR) = 1 were set at clinically meaningful points: the median value for each variable, or at a previously validated clinical threshold when available from the literature. A multivariable logistic regression model incorporating the selected predictors (with spline or linear terms as determined above) was subsequently constructed, and a nomogram was derived to estimate the individualized probability of MPNP.

### 2.4 Model validation

Model performance was evaluated for discrimination, calibration, and clinical utility. Discrimination was quantified by the area under the receiver operating characteristic curve (AUC) with 95% confidence intervals. Calibration was assessed using calibration plots comparing predicted and observed probabilities. Clinical utility was examined by decision curve analysis (DCA) across a range of threshold probabilities. All three metrics were internally validated using 1,000 bootstrap resamples to correct for optimism.

### 2.5 Statistical software

All analyses were performed in R (version 4.6.0; R Foundation for Statistical Computing, Vienna, Austria). The glmnet package was used for LASSO regression, and the rms package was used for restricted cubic spline modeling, nomogram construction, and model validation. A two-sided *P* < 0.05 was considered statistically significant.

## 3 Results

### 3.1 Baseline characteristics of the study population

A total of 319 children hospitalized with *Mycoplasma pneumoniae* pneumonia met the eligibility criteria, of whom 146 (45.8%) were classified into the MPNP group and 173 (54.2%) into the MPNNP group (Table 1).

**Table 1:** Baseline demographic, clinical, and inflammatory characteristics of children in the MPNP and MPNNP groups.

Variables	MPNP group (*n* = 146)	MPNNP group (*n* = 173)	*P* value
Male sex, *n* (%)	74 (50.7)	80 (46.2)	0.429
Age, years	8 (6, 11)	8 (6, 11)	0.255
Weight, kg	28.4 (22.2, 39.2)	31.3 (21.9, 39.8)	0.242
Height, cm	113.9 (98.9, 125.3)	113.6 (99.0, 128.2)	0.550
Peak temperature, °C	38.8 (38.4, 39.2)	38.8 (38.5, 39.0)	0.942
Fever duration, days	14 (11, 16)	9 (6, 11)	<0.001
Cough duration, days	17 (13, 21)	18 (14, 23)	0.077
Eczema history, *n* (%)	11 (7.5)	12 (6.9)	0.837
Rhinitis history, *n* (%)	8 (5.5)	15 (8.7)	0.272
Abnormal lung auscultation, *n* (%)	146 (100.0)	173 (100.0)	1.000
Unilateral involvement, *n* (%)	101 (69.2)	120 (69.4)	0.971
Atelectasis, *n* (%)	25 (17.1)	41 (23.7)	0.149
Pleural effusion, *n* (%)	83 (56.8)	93 (53.8)	0.580
Decreased breath sounds, *n* (%)	93 (63.7)	101 (58.4)	0.332
WBC, ×10⁹/L	19.76 (15.34, 23.57)	12.10 (9.53, 15.14)	<0.001
Neutrophil, %	74.4 (69.2, 79.7)	73.3 (67.5, 79.6)	0.518
CRP, mg/L	82.3 (57.2, 113.3)	46.4 (31.9, 77.1)	<0.001
Hemoglobin, g/L	129.0 (120.3, 136.3)	125.6 (118.8, 134.9)	0.322
Platelet, ×10⁹/L	283 (236, 343)	295 (241, 343)	0.445
Procalcitonin, ng/mL	0.13 (0.08, 0.20)	0.15 (0.08, 0.22)	0.222
Lactic acid, mmol/L	2.46 (2.20, 2.80)	2.49 (2.19, 2.81)	0.725
Prealbumin, g/L	0.11 (0.08, 0.15)	0.12 (0.08, 0.18)	0.194
LDH, U/L	314 (260, 397)	323 (267, 393)	0.574
AST, U/L	22 (19, 28)	22 (18, 27)	0.818
ALT, U/L	13 (10, 16)	14 (11, 19)	0.085
CK, U/L	89 (65, 116)	85 (64, 108)	0.369
CK-MB, U/L	9 (7, 10)	8 (7, 10)	0.109
IL-6, pg/mL	50.54 (36.24, 80.77)	51.93 (35.80, 76.88)	0.902
IgE, IU/mL	134.38 (74.52, 221.19)	127.26 (78.42, 226.45)	0.603
Ferritin, μg/L	274.38 (185.92, 414.28)	295.29 (220.38, 452.03)	0.093
ESR, mm/h	26 (18, 34)	27 (20, 40)	0.176
D-dimer, mg/L	0.52 (0.37, 0.73)	0.49 (0.36, 0.74)	0.657
Fibrinogen, g/L	4.07 (3.29, 4.66)	4.14 (3.42, 4.81)	0.395

The two groups were well balanced with respect to baseline demographic characteristics, including sex (male, 50.7% vs. 46.2%; *P* = 0.429), age median [IQR], 8 [6–11] vs. 8 [6–11] years; *P* = 0.255), body weight (*P* = 0.242), and height (*P* = 0.550). With regard to clinical presentation, fever duration was markedly longer in the MPNP group than in the MPNNP group [median [IQR], 14 [11–16] vs. 9 [6–11] days; *P* < 0.001]. In contrast, peak temperature (*P* = 0.942), cough duration (*P* = 0.077), and histories of eczema (*P* = 0.837) and rhinitis (*P* = 0.272) did not differ significantly between groups. All children had abnormal pulmonary auscultation, and the frequencies of unilateral involvement (69.2% vs. 69.4%; *P* = 0.971), atelectasis (17.1% vs. 23.7%; *P* = 0.149), pleural effusion (56.8% vs. 53.8%; *P* = 0.580), and diminished breath sounds (63.7% vs. 58.4%; *P* = 0.332) were comparable.

Among the 19 laboratory indices evaluated, only two differed significantly between groups. Children with MPNP exhibited substantially higher WBC counts [median [IQR], 19.76 [15.34–23.57] vs. 12.10 [9.53–15.14] × 10⁹/L; *P* < 0.001] and CRP concentrations [median [IQR], 82.3 [57.2–113.3] vs. 46.4 [31.9–77.1] mg/L; *P* < 0.001]. The remaining markers, including neutrophil percentage (*P* = 0.518), procalcitonin (*P* = 0.222), lactate dehydrogenase (*P* = 0.574), interleukin-6 (*P* = 0.902), ferritin (*P* = 0.093), D-dimer (*P* = 0.657), and fibrinogen (*P* = 0.395), showed no significant intergroup differences.

### 3.2 Feature selection by LASSO regression

To minimize overfitting and address potential multicollinearity, 32 of the 33 collected variables were entered into a LASSO logistic regression model. Abnormal pulmonary auscultation was excluded from modeling because it was present in 100% of children in both groups (i.e., a constant with zero variance), rendering it uninformative for discriminating between MPNP and MPNNP. As the penalty coefficient (*λ*) increased, the coefficients of progressively more variables shrank toward zero (Figure 1A). The optimal *λ* was determined by 10-fold cross-validation under the minimum binomial deviance criterion (Figure 1B). At the optimal *λ*, three variables retained non-zero coefficients and were identified as the most informative predictors of MPNP: WBC count, CRP concentration, and fever duration. These results were concordant with the univariable comparisons in Table 1.

**Figure 1:**
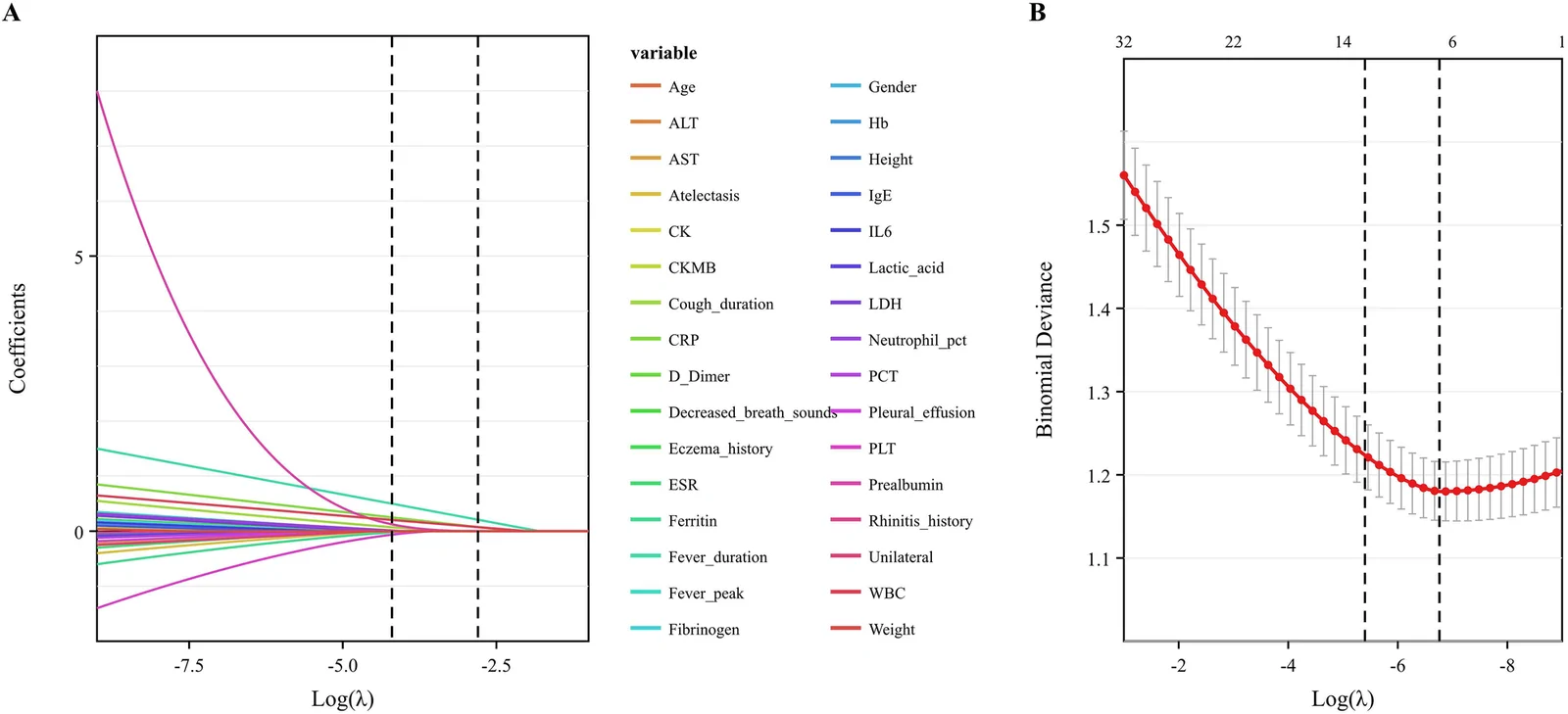
LASSO logistic regression for feature selection. **(A)** Coefficient profiles of the 32 candidate variables across log(*λ*). **(B)** Optimal *λ* selected by 10-fold cross-validation based on minimum binomial deviance; dashed lines indicate *λ*.min and *λ*.1se. Variables with non-zero coefficients at the optimal *λ* were retained.

### 3.3 Non-linear dose–response relationships

Following the pre-specified screening procedure described in Section 2.3, RCS analyses were applied to all three LASSO-selected continuous predictors to formally evaluate the presence of non-linear associations.

For CRP, both the overall association (*P* < 0.001) and the non-linearity component (*P* < 0.001) were statistically significant, indicating a significant non-linear relationship with MPNP risk. AIC comparison favored a four-knot specification (knots at the 5th, 35th, 65th, and 95th percentiles, corresponding to approximately 20, 47, 80, and 147 mg/L), which provided two non-linear degrees of freedom to accommodate the observed non-monotonic trajectory. With the reference value set at the median CRP (65 mg/L, OR = 1), the adjusted OR rose steeply as CRP increased from low to intermediate concentrations, reaching a peak at approximately 100–130 mg/L, and subsequently declined at very high CRP levels, describing an inverted U-shaped dose–response pattern (Figure 2). This non-monotonic relationship suggests that the incremental risk of MPNP associated with CRP elevation is concentrated within the intermediate range, while very high CRP values may not confer proportionally greater risk.

**Figure 2:**
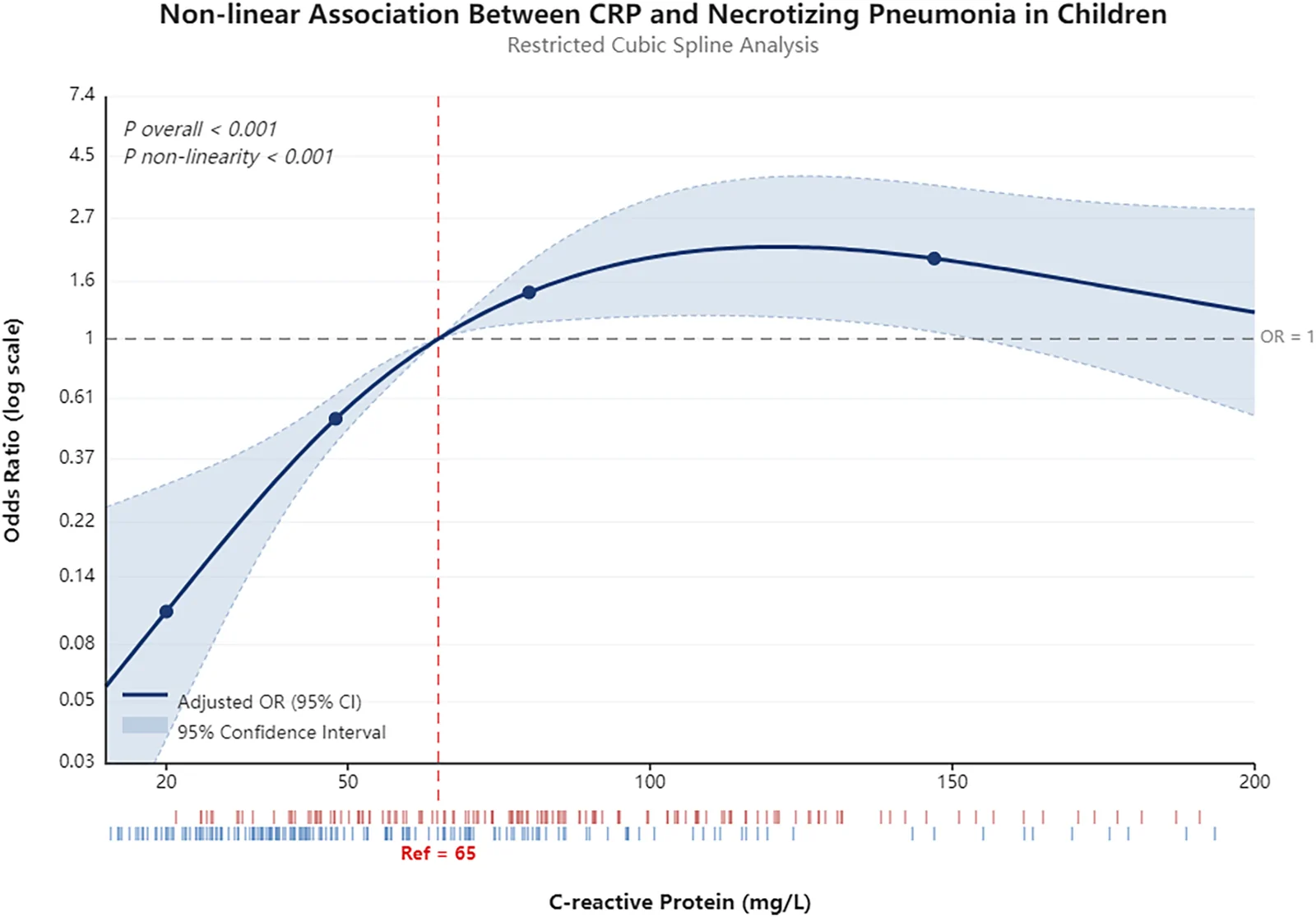
Restricted cubic spline analysis of the association between C-reactive protein (CRP) and the risk of *Mycoplasma pneumoniae*-associated necrotizing pneumonia. The solid line and shaded area represent the adjusted odds ratio (OR) and 95% CI; the reference value (OR = 1) was set at the median CRP (red dashed line). *P*-overall and *P*-non-linearity are shown.

For WBC count, the overall association was significant (*P* < 0.001) and the non-linearity test also reached significance (*P* = 0.014), confirming a non-linear relationship. AIC comparison favored a three-knot specification (knots at the 10th, 50th, and 90th percentiles, corresponding to approximately 6.0, 12.3, and 21 × 10⁹/L), providing one non-linear degree of freedom that adequately captured the predominantly monotonic but saturating association. The reference value was set at 12.3 × 10⁹/L based on a clinically established threshold reported in prior literature (7). The risk of MPNP increased sharply across the lower-to-intermediate range of WBC counts, crossing the null value (OR = 1) at approximately the reference point, and subsequently attenuated to a plateau at higher counts (Figure 3). This threshold-like behavior indicated that the incremental risk conferred by rising WBC was concentrated within a critical intermediate range.

**Figure 3:**
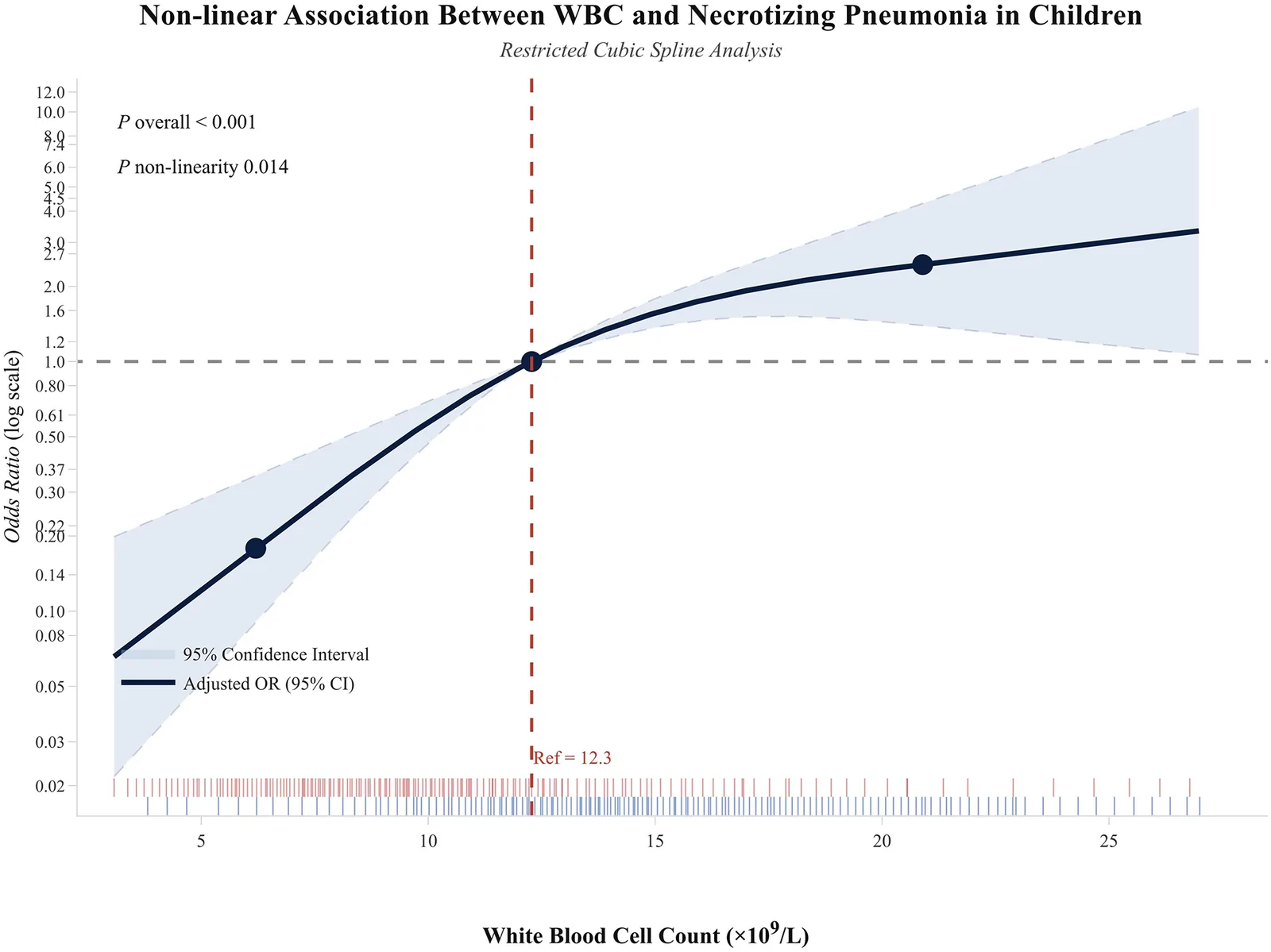
Restricted cubic spline analysis of the association between white blood cell (WBC) count and the risk of *Mycoplasma pneumoniae*-associated necrotizing pneumonia. The solid line and shaded area represent the adjusted OR and 95% CI; the reference value (OR = 1) was set at 12.3 × 10⁹/L based on prior literature (Zheng et al., 2020) (red dashed line). *P*-overall and *P*-non-linearity are shown.

For fever duration, the overall association was significant (*P* < 0.001); however, the non-linearity test was not significant (*P* = 0.372), indicating that the relationship between fever duration and MPNP risk was adequately described by a linear term. Accordingly, fever duration was entered into the final multivariable model as a continuous linear predictor.

### 3.4 Development of the predictive nomogram

A multivariable logistic regression model incorporating CRP and WBC as restricted cubic splines together with fever duration as a linear term was constructed, and the resulting coefficients were translated into a graphical nomogram for individualized estimation of MPNP probability (Figure 4). Each predictor was assigned a point score; the summed total points corresponded to an estimated risk on the lowest scale. In a representative worked example, a patient accumulating approximately 215 total points corresponded to a predicted MPNP probability of approximately 0.83, illustrating the practical bedside application of the model.

**Figure 4:**
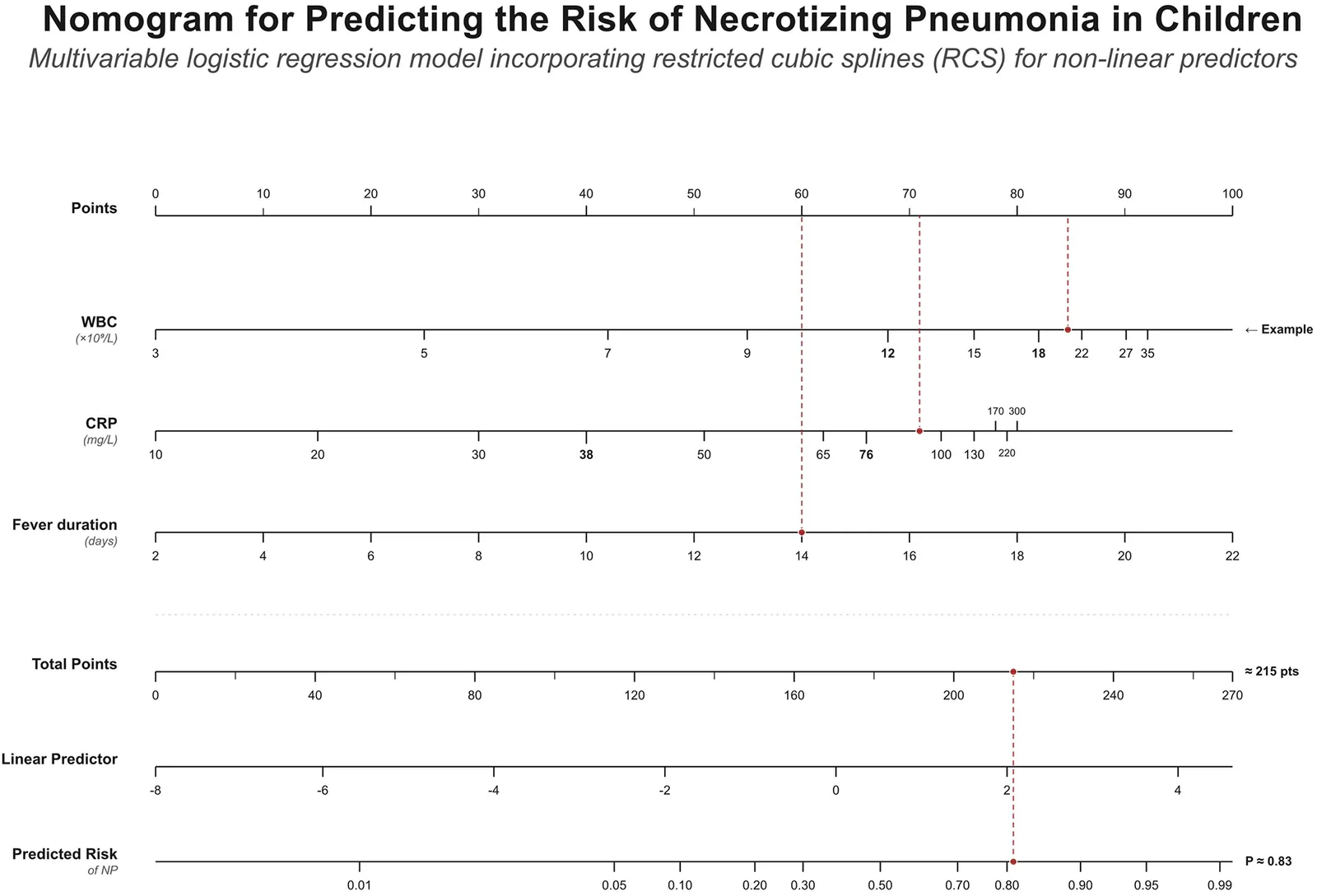
Nomogram for predicting the risk of *Mycoplasma pneumoniae*-associated necrotizing pneumonia, incorporating WBC, CRP, and fever duration with restricted cubic splines for non-linear predictors. The dashed red line shows a worked example (total points ≈ 215, predicted risk ≈ 0.83).

### 3.5 Model performance and internal validation

#### 3.5.1 Discrimination

The nomogram showed strong discriminative ability in the derivation cohort, with an apparent AUC of 0.941 (95% CI, 0.908–0.975) (Figure 5A). After internal validation using 1,000 bootstrap resamples to correct for optimism, the model retained strong performance, with a bias-corrected AUC of 0.920 (95% CI, 0.877–0.962) (Figure 5B).

**Figure 5:**
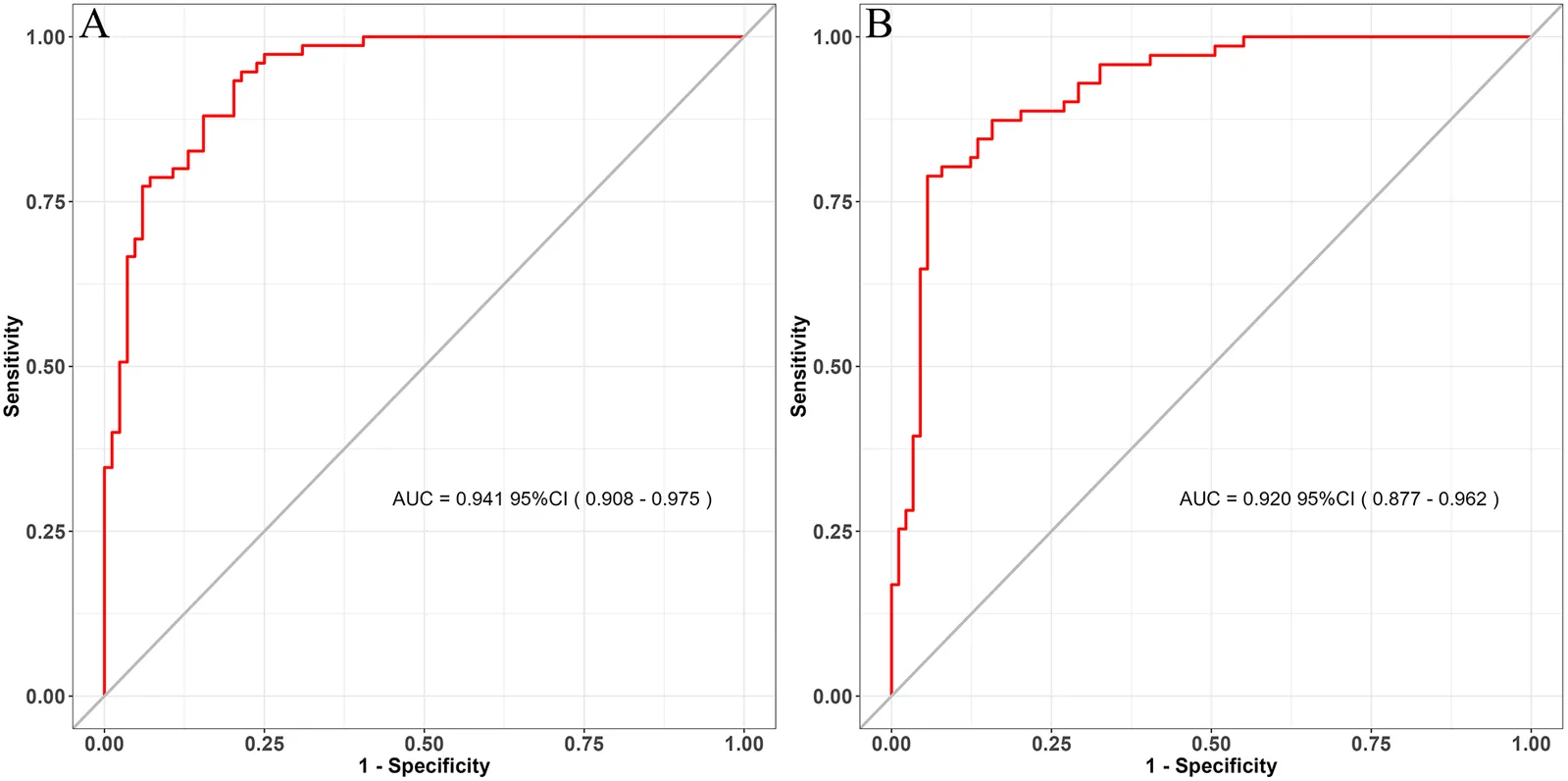
ROC curves of the model. **(A)** Apparent performance (AUC = 0.941, 95% CI: 0.908–0.975) and **(B)** bootstrap-corrected performance after 1,000 resamples (AUC = 0.920, 95% CI: 0.877–0.962).

#### 3.5.2 Calibration

The calibration curves demonstrated close agreement between predicted and observed probabilities of MPNP. Calibration. The apparent calibration curve (Figure 6A) demonstrated close agreement between predicted and observed probabilities of MPNP, tracking the 45° reference line across most of the predicted risk spectrum with only minor deviations at higher predicted probabilities. The bootstrap-corrected calibration curve (Figure 6B) revealed modest underestimation of observed risk in the low-to-moderate predicted probability range (approximately 0.05–0.40), with improved agreement at higher predicted probabilities. Overall, the calibration performance remained acceptable, indicating that the nomogram provides reasonably accurate probability estimates, though slight recalibration may further improve predictions in the intermediate-risk zone. Clinical utility. DCA confirmed the clinical value of the nomogram. Across a broad range of threshold probabilities, the model yielded a greater net benefit than either the “treat-all” or “treat-none” strategies (Figure 7A), and this advantage was preserved after bootstrap correction (Figure 7B), supporting the robustness and clinical applicability of the model for risk-stratified decision making.

**Figure 6:**
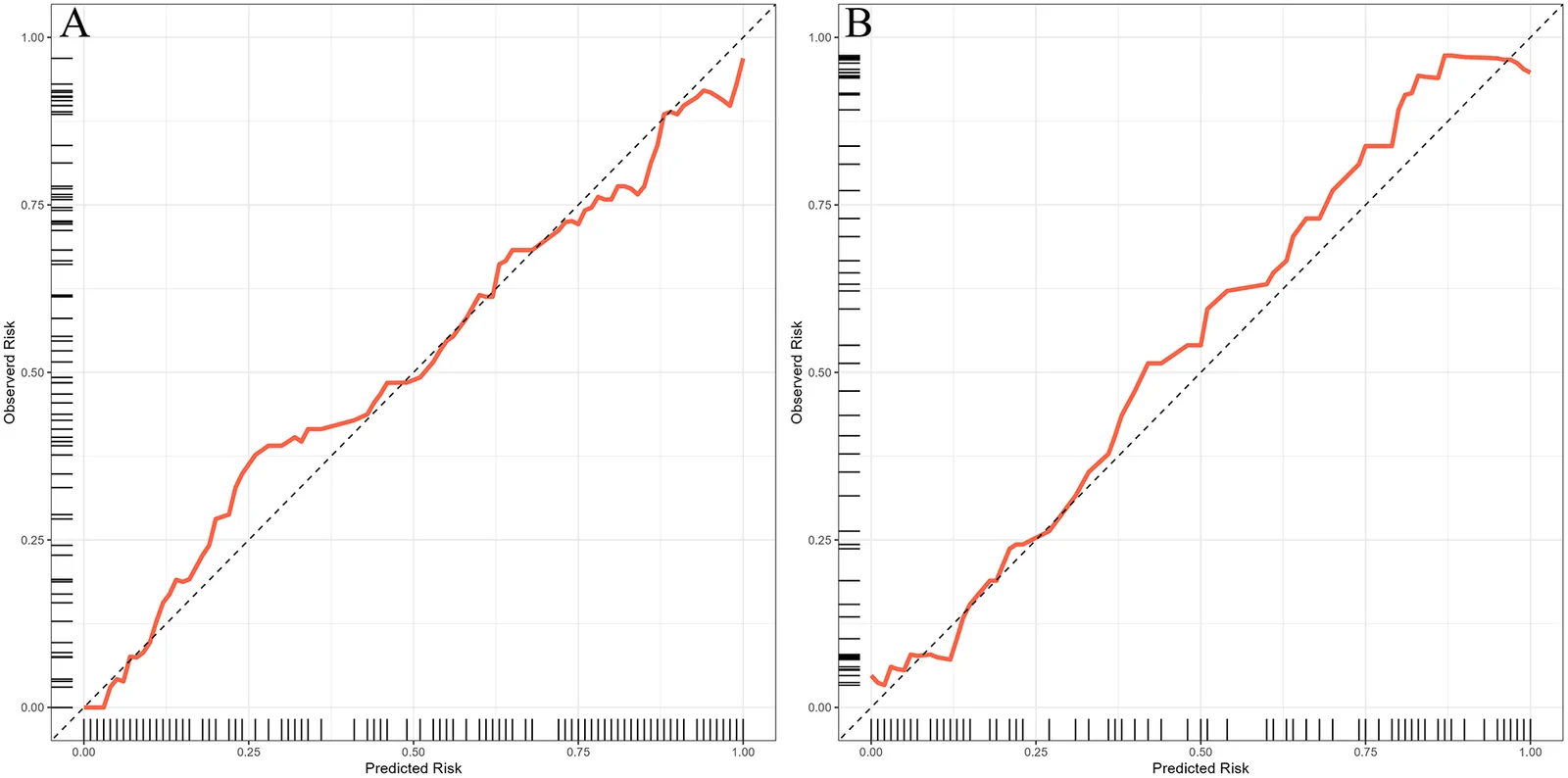
Calibration curves of the nomogram. **(A)** Apparent and **(B)** bootstrap-corrected (1,000 resamples) performance. The diagonal dashed line indicates perfect agreement; the solid line represents model calibration.

**Figure 7:**
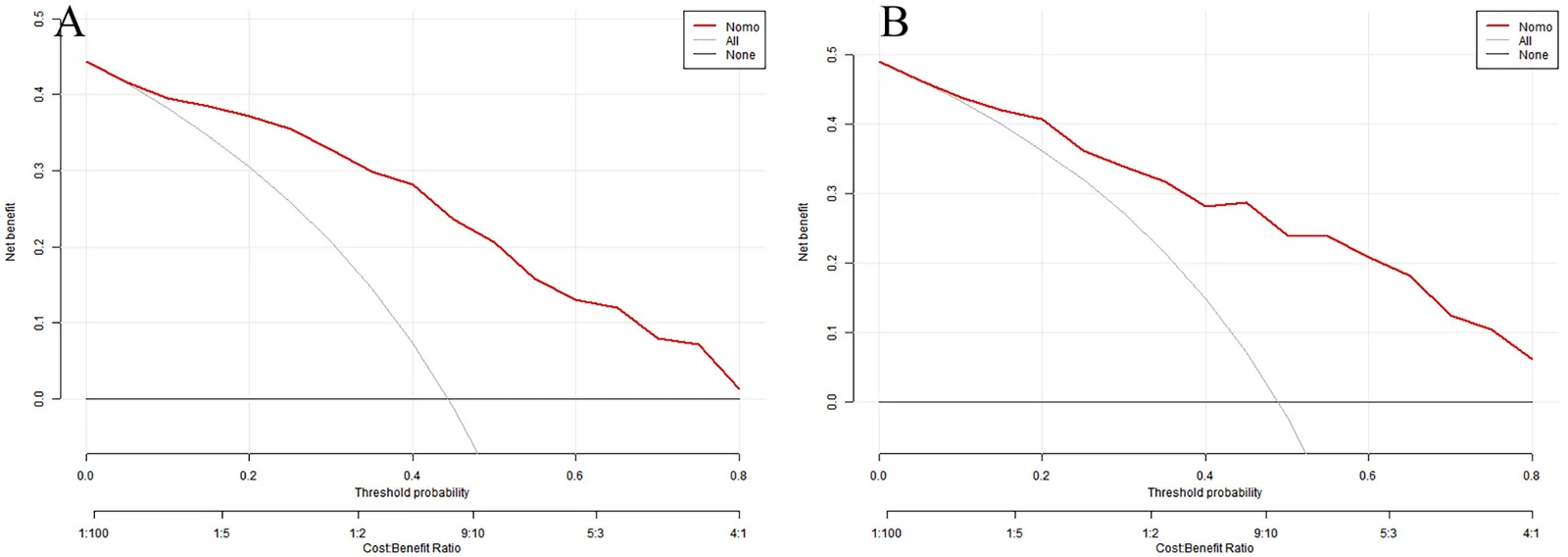
Decision curve analysis of the nomogram. **(A)** Apparent and **(B)** bootstrap-corrected performance. “Nomo,” prediction model; “All,” treat-all strategy; “None,” treat-none strategy.

Taken together, these results demonstrate that fever duration, WBC count, and CRP concentration—two of which exhibited distinct non-linear associations with disease risk (a threshold-like pattern for WBC and an inverted U-shaped pattern for CRP)—independently identify children at high risk of MPNP, and that a parsimonious RCS-based nomogram integrating these three readily available predictors provides favorable discrimination, acceptable calibration, and potential clinical utility in this single-center derivation cohort, pending external validation.

## 4 Discussion

Necrotizing pneumonia is an increasingly recognized and clinically significant complication of MPP in children, yet its diagnosis is typically delayed, as contrast-enhanced chest CT reveals characteristic parenchymal necrosis a median of 16–25 days after symptom onset (4, 7). This diagnostic lag may result in missed opportunities for timely therapeutic intervention. In the present study, we applied RCS analysis—for the first time in the context of MPNP—to characterize the non-linear dose–response relationships between key inflammatory biomarkers and disease risk, and subsequently developed and internally validated a parsimonious nomogram integrating three readily obtainable clinical variables: WBC count, CRP concentration, and fever duration. Our RCS-based nomogram demonstrated strong internal discrimination (apparent AUC = 0.941; bootstrap bias-corrected AUC = 0.920), acceptable calibration, and potential net clinical benefit across a broad range of decision thresholds, although these findings require confirmation in independent cohorts. These performance metrics compare favorably with those of previously reported linear prediction models. Luo and Wang (9) constructed a LASSO-based nomogram incorporating bacterial co-infection, chest pain, LDH, CRP, fever duration, and D-dimer, achieving AUCs of 0.870 and 0.843 in their training and validation sets, respectively. Li et al. (10) developed a four-predictor nomogram (fever duration, WBC count, IL-6, and D-dimer) with a validation AUC of 0.920. Zhang et al. (5) identified age, fever days, D-dimer, LDH, and pleural effusion as independent risk factors (combined AUC = 0.940). More recently, Ren et al. (16) applied dose–response analysis and confirmed a non-linear relationship between LDH and MPNP incidence. In contrast to these models, our approach achieves comparable or superior discrimination using only three routine clinical and laboratory variables while, crucially, accounting for non-linear risk trajectories—an advance that addresses a recognized methodological limitation of earlier studies that uniformly imposed linear assumptions on continuous predictors (15, 17).

The identification of WBC count as an independent predictor of MPNP, exhibiting a distinct non-linear, threshold-like dose–response relationship (*P*-non-linearity = 0.014), constitutes a key finding of this study. The RCS curve demonstrated that MPNP risk rose sharply across the lower-to-intermediate range of WBC counts and subsequently plateaued at higher values, suggesting that the incremental risk conferred by rising WBC is concentrated within a critical intermediate zone. This non-linear pattern is biologically plausible and aligns with the current understanding of MPNP pathogenesis. Upon *M. pneumoniae* infection, robust activation of the innate immune system triggers the release of pro-inflammatory cytokines such as IL-1β, IL-6, IL-8, and TNF-α through TLR2/TLR4-mediated NF-*κ*B signaling pathways, resulting in neutrophil recruitment and amplification of pulmonary inflammation (6, 18). The initial steep rise in risk likely reflects the transition from a contained inflammatory response to an immune-overactivation state characterized by neutrophil-mediated tissue damage. The subsequent plateau suggests that beyond a certain degree of leukocytosis, additional increments in WBC offer diminishing prognostic information, possibly because the immunopathological cascade—including local vasculitis, coagulation activation, and microthrombus formation—has already been fully initiated (7, 19). Zheng et al. (7) previously identified WBC > 12.3 × 10⁹/L as an independent risk factor for MPNP (OR = 6.412). We adopted this clinically validated threshold as the reference point (OR = 1) for our RCS analysis, and the spline curve confirmed that risk rose sharply below this value and plateaued above it, thereby corroborating the biological significance and clinical utility of this threshold. Importantly, the non-linear modeling approach avoids the information loss inherent in dichotomizing WBC at an arbitrary cut-off, instead capturing the full gradient of risk across the continuum—an advantage increasingly recognized in clinical epidemiology (17, 20).

CRP, an acute-phase reactant synthesized by hepatocytes predominantly in response to IL-6 and other pro-inflammatory stimuli, demonstrated a strong overall association with MPNP risk (*P*-overall < 0.001; *P*-non-linearity < 0.001) characterized by an inverted U-shaped dose–response curve. Risk increased steeply through the lower and intermediate CRP ranges, peaked at approximately 100–130 mg/L, and subsequently declined at very high concentrations. Interestingly, our inverted U-shaped pattern differs from the saturating relationship between CRP and mortality reported in COVID-19 patients (21), suggesting disease-specific non-linear risk profiles. The decline in OR at very high CRP levels is a novel finding that warrants further investigation in independent cohorts. Li et al. (21) demonstrated a curved relationship and saturation effect between CRP and mortality in 3,545 SARS-CoV-2–infected patients, showing that when CRP was below 15.6 mg/L, mortality increased significantly with each 10 mg/L increment (adjusted HR = 1.57), whereas above this inflection point the rate of increase markedly attenuated (HR = 1.11). Similarly, a large meta-analysis of cohort studies by Li et al. (22) confirmed that the relationships between CRP and all-cause and cardiovascular mortality were non-linear with a saturation pattern, while cancer mortality was linearly related to CRP. In the specific context of MPNP, CRP elevation reflects the magnitude of the systemic inflammatory response driven by host immune overreaction to *M. pneumoniae* (18, 19). Zhou et al. (23) demonstrated that CRP was significantly higher in children with MPNP compared with non-necrotizing MPP, and Luo and Wang (9) similarly identified CRP as an independent predictor. The inverted *U*-shaped pattern observed in our study can be interpreted through recent insights into CRP conformational biology and its immunomodulatory duality. CRP circulates predominantly as a pentameric isoform (pCRP), which recruits complement factor H to damaged cell surfaces and interacts with C4b-binding protein, thereby limiting complement overactivation and restricting bystander tissue injury (24–26). In contrast, monomeric CRP (mCRP), generated by irreversible dissociation of pCRP at sites of active inflammation, potently activates the classical complement pathway and drives neutrophil extracellular trap (NET) formation, amplifying local tissue destruction (27). At very high circulating CRP concentrations, the sustained predominance of the anti-inflammatory pCRP isoform in the systemic compartment may partially counterbalance the pro-inflammatory effects of locally generated mCRP, providing a plausible molecular mechanism for the attenuated incremental risk observed beyond approximately 130 mg/L. Second, extremely high CRP values may reflect superimposed bacterial co-infection rather than the *Mycoplasma*-driven inflammatory cascade primarily responsible for MPNP. Procalcitonin (PCT) can aid in this distinction, as it remains characteristically low in isolated atypical infections but rises substantially with bacterial superinfection (28). In our cohort, PCT did not differ between groups (*P* = 0.222), suggesting a predominantly *Mycoplasma*-driven response overall; however, we did not perform PCT-stratified analysis specifically within the extreme-CRP subgroup, and this hypothesis warrants targeted investigation in future studies. Third, the wide confidence intervals at the extreme right tail of the CRP distribution reflect relatively sparse data in this range, and the observed decline in OR should be interpreted with caution. Clinically, this non-linear pattern suggests that moderate CRP elevations (50–130 mg/L) confer the highest incremental risk of MPNP and should prompt heightened vigilance, whereas very high CRP values (>150 mg/L) may not indicate proportionally greater MPNP risk and could warrant evaluation for bacterial co-infection using PCT and microbiological testing.

Fever duration emerged as the third independent predictor and displayed an approximately linear, positive association with MPNP risk. Each additional day of fever was associated with a progressive increase in the odds of developing necrotizing pneumonia. This finding is highly consistent with the existing literature: multiple studies have identified prolonged fever as a significant risk factor for MPNP (5, 7, 9, 10). Zhang et al. (5) reported that fever duration >7.5 days predicted MPNP with high sensitivity and specificity. In our cohort, the MPNP group exhibited a median fever duration of 14 days versus 9 days in the non-necrotizing group (*P* < 0.001). Prolonged fever is generally considered a clinical surrogate for sustained and excessive inflammatory activation. He et al. (6) demonstrated that the deterioration of MPP is driven primarily by an excessive neutrophil-mediated immune response rather than by pathogen burden *per se*, implying that the duration of immune overactivation—clinically manifested as persistent fever—directly determines the extent of parenchymal necrosis. A recent study further elucidated that immune dysregulation in MPP is characterized by excessive cytokine release and abnormal activation of innate and adaptive immune cells during the acute phase, with the inflammatory storm perpetuating tissue destruction over time (3). Importantly, unlike WBC and CRP, which require laboratory testing, fever duration is a purely clinical variable that can be assessed at the bedside without additional cost, thereby enhancing the accessibility and applicability of our model in resource-constrained settings.

Several methodological strengths of the present study merit emphasis. First, the application of LASSO regression for variable selection from 32 candidate predictors minimized overfitting and multicollinearity, ensuring that only the most informative variables were retained. Second, the integration of RCS analysis into the logistic regression framework preserved model interpretability while capturing the non-linear dose–response relationships that traditional linear models would have missed. As Schuster et al. (17) demonstrated, RCS models consistently outperform both categorization and linear approaches in explained variance and prediction accuracy; RCS regression is particularly recommended when the aim is individualized prediction—precisely the purpose of our nomogram. Third, our comprehensive internal validation strategy using 1,000 bootstrap resamples with bias correction for the AUC, calibration curves, and decision curve analysis provides robust evidence of the model's stability and generalizability.

Nevertheless, this study has several limitations. First, this is a retrospective, single-center study restricted to inpatients, meaning that milder MPNNP cases managed in outpatient settings were not captured; the near-equal group sizes (146 vs. 173) thus reflect referral-based sampling rather than population prevalence, and the model's performance in lower-acuity settings remains to be determined. Second, all modeling steps were performed on the same cohort; although 1,000 bootstrap resamples were used to correct for optimism, this cannot replace independent external validation, and the predictive stability of the nomogram remains to be confirmed in multicenter cohorts as recommended by the TRIPOD guidelines (29). Third, the study period (2020–2025) overlapped with the COVID-19 pandemic and the subsequent *M. pneumoniae* resurgence attributed to “immunity debt” (30, 31), which may have introduced confounding heterogeneity. Prolonged absence of *M. pneumoniae* circulation during non-pharmaceutical interventions likely expanded the immunologically naïve pediatric population, potentially amplifying host inflammatory responses upon re-exposure and shifting the baseline immune status of susceptible children. Concurrently, changes in circulating *M. pneumoniae* genotypes or macrolide resistance profiles during the post-pandemic resurgence may have influenced pathogen virulence and disease severity. These factors could affect the absolute values of the dose–response thresholds identified herein and may limit direct generalizability to non-pandemic periods. Nonetheless, the three predictors retained by our model—WBC count, CRP, and fever duration—have been consistently identified across both pre-pandemic (7, 23) and pandemic-era (5, 9, 10) studies, suggesting that the underlying immunopathological relationships driving MPNP risk are likely robust across epidemiological contexts, even if the precise inflection points may require recalibration in future cohorts. Fourth, potentially relevant immunological biomarkers such as T-cell subsets and Th1/Th2 cytokine profiles were not routinely available and could not be evaluated (3). Fifth, laboratory values were obtained from a single admission blood sample and may not capture the dynamic trajectory of inflammatory markers during disease progression. Finally, the present study focused on characterizing non-linear dose–response relationships and continuous probability estimation; determining an optimal cutoff for the composite nomogram score and comparing its stratification performance with single-biomarker thresholds would require external validation in independent multicenter cohorts and represents an important direction for future research.

In conclusion, WBC count, CRP, and fever duration independently predict MPNP in children, with WBC and CRP exhibiting distinct non-linear dose–response patterns not previously characterized in this context. The RCS-based nomogram integrating these three readily available predictors demonstrates promising predictive performance in internal validation; however, multicenter external validation is essential to confirm its accuracy and clinical utility before implementation in routine practice.

## Data Availability

The original contributions presented in the study are included in the article; further inquiries can be directed to the corresponding author upon reasonable request. Requests to access these datasets should be directed to zhengjiafeng001@126.com.
